# Three-dimensional bright-field microscopy with isotropic resolution based on multi-view acquisition and image fusion reconstruction

**DOI:** 10.1038/s41598-020-69730-4

**Published:** 2020-07-29

**Authors:** Gianmaria Calisesi, Alessia Candeo, Andrea Farina, Cosimo D’Andrea, Vittorio Magni, Gianluca Valentini, Anna Pistocchi, Alex Costa, Andrea Bassi

**Affiliations:** 1 Dipartimento di Fisica, Politecnico di Milano, piazza Leonardo da Vinci 32, 20133 Milan, Italy; 2 Istituto di Fotonica e Nanotecnologie, Consiglio Nazionale delle ricerche, piazza Leonardo da Vinci 32, 20133 Milan, Italy; 3 Dipartimento di Biotecnologie Mediche e Medicina Traslazionale, Università degli Studi di Milano, via Fratelli Cervi 93, 20090 Segrate, Italy; 4 Dipartimento di Bioscienze, Università degli Studi di Milano, via Celoria 26, 20133 Milan, Italy

**Keywords:** Light-sheet microscopy, Wide-field fluorescence microscopy, Biophotonics

## Abstract

Optical Projection Tomography (OPT) is a powerful three-dimensional imaging technique used for the observation of millimeter-scaled biological samples, compatible with bright-field and fluorescence contrast. OPT is affected by spatially variant artifacts caused by the fact that light diffraction is not taken into account by the straight-light propagation models used for reconstruction. These artifacts hinder high-resolution imaging with OPT. In this work we show that, by using a multiview imaging approach, a 3D reconstruction of the bright-field contrast can be obtained without the diffraction artifacts typical of OPT, drastically reducing the amount of acquired data, compared to previously reported approaches. The method, purely based on bright-field contrast of the unstained sample, provides a comprehensive picture of the sample anatomy, as demonstrated in vivo on *Arabidopsis thaliana* and zebrafish embryos. Furthermore, this bright-field reconstruction can be implemented on practically any multi-view light-sheet fluorescence microscope without complex hardware modifications or calibrations, complementing the fluorescence information with tissue anatomy.

## Introduction

Three-dimensional optical imaging techniques are essential tools for observing the structure and understanding the function of biological samples. Among them Optical Projection Tomography (OPT), is well suited to study specimens ranging in size from hundreds of microns to a centimeter^1^. OPT is often considered the optical analogous of X-ray Computed Tomography (CT), performing tomographic optical imaging of three dimensional samples with transmitted and fluorescent light. OPT can be used in a number of different applications with specimens that include embryos^2,3^, mouse organs^4–6^ and plants^7^. At the same time novel OPT configurations have been presented to achieve fast acquisition^8–10^, to reconstruct the fluorescence lifetime and Förster resonance energy transfer contrast^11,12^, to obtain the contrast from blood flow^13–16^. In parallel, advanced recontruction algorithms have constantly been developed^17–19^.

Remarkably, transmission OPT, which provides bright-field contrast, is used to correct absorption artifacts in Light Sheet Fluorescence Microscopy (LSFM)^20,21^ and for multimodal reconstruction of the whole specimen’s anatomy^22^.

The idea behind OPT is to acquire images (or projections) of the sample from different orientations. Similarly to X-ray CT the sample is then reconstructed using a back-projection algorithm^23^. However, this algorithm assumes that the light beam propagates straight through the sample: in X-ray CT the straight propagation is given by the high frequency of the radiation, whereas in OPT we can consider a straight propagation of light only within the depth of field of the system. Since the depth of field scales with the inverse of the second power of the Numerical Aperture (*NA*), the assumption is valid only at low *NA* (c.a < 0.1) which typically corresponds to a low magnification (1×*, *2×). At higher *NA*s, artifacts due to the diffraction of light are present^24^. One way to attenuate these artifacts consists in limiting the NA of the microscope (e.g. by inserting a diaphragm at the back focal plane of the detection objective), but this also limits the resolution. Another way to mitigate this effect is to incorporate a spatially variant deconvolution in the reconstruction algorithm^24,25^. To drastically remove the diffraction artifact, some methods based on the extension of the depth of field have been proposed^21,26–28^. For each projection, the sample is scanned at different positions (up to 100) along the optical axis, and the acquired stack of images is merged into a single entirely focused projection^29^. Yet, this come at the expenses of the number of acquired images, since the process must be repeated for each projection (up to 1,000 angles within 360° rotation).

Here we show that the Bright-Field (BF) contrast can be efficiently reconstructed in 3D by adopting a multi-view image processing method: we propose to fuse three-dimensional image data sets of the sample acquired at multiple angles into a single reconstruction. Each data set consists of a stack of bright-field images acquired by scanning the sample along the optical axis. The method requires the acquisition of a reduced number of angles and eliminates the diffraction artifacts, still providing isotropic resolution and 3D reconstruction of unlabeled samples. The reconstruction is not based on a back-projection algorithm, it uses a multiview fusion approach, that is frequently used in fluorescence imaging^30^. Here we describe how to apply this approach to reconstruct the BF contrast and we identify the optimal parameters for reconstruction as a function of the spatial and angular sampling. We present *in-vivo* data of *Arabidopsis thaliana* and zebrafish (*Danio rerio*) in order to demonstrate that the approach allows one to observe the whole anatomy of unstained organisms. Finally, we show that, with small modification of the hardware, BF reconstruction can be readily implemented in any multi-view Light Sheet Fluorescence Microscope (LSFM)^31,32^, obtaining multimodal (bright-field and fluorescence) acquisitions with the same instrument.

## Results and discussion

### Bright-field multi-view reconstruction

**Figure 1:**
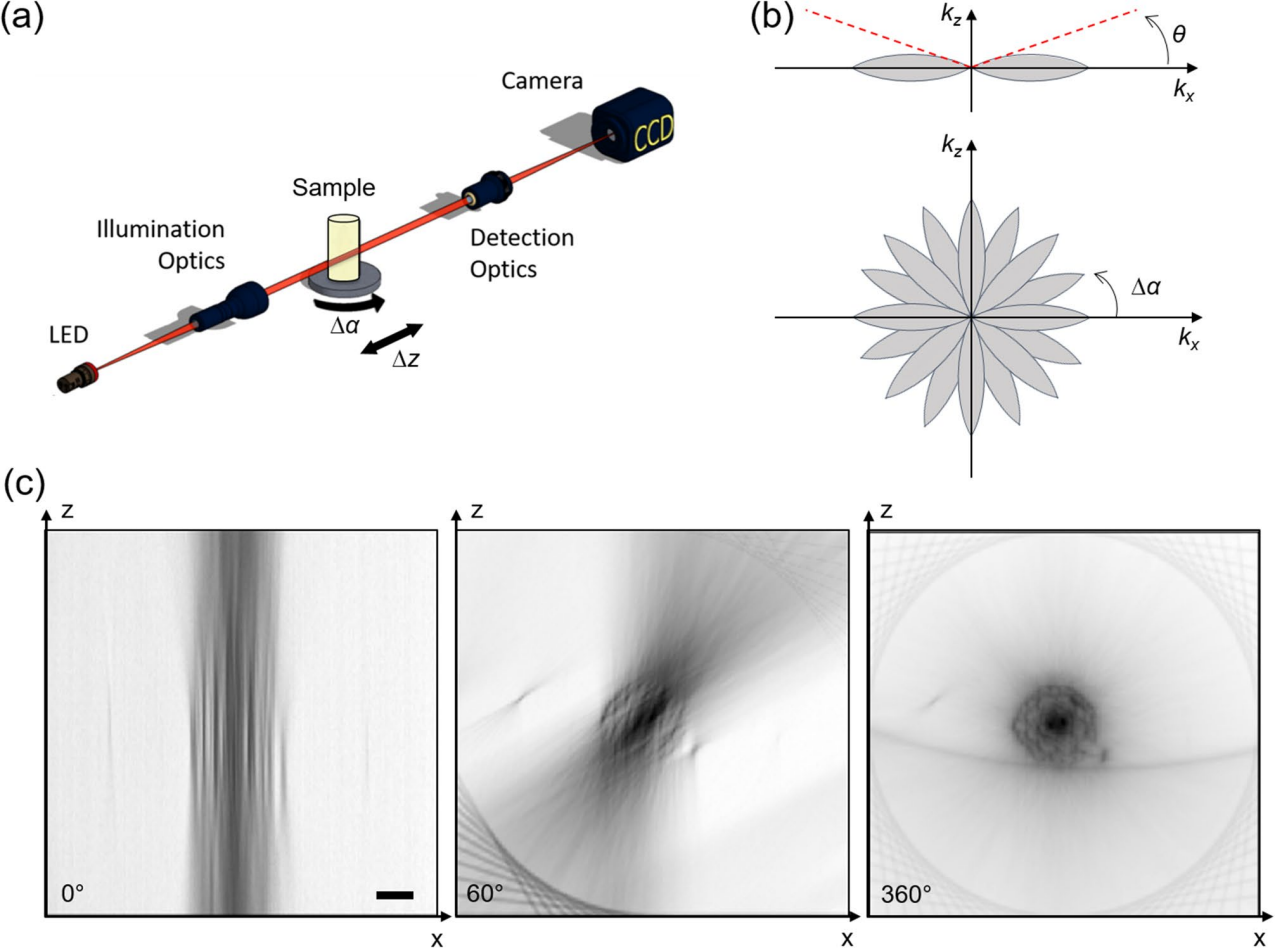
Experimental setup for multi-view bright-field reconstruction: an LED (530 nm) illuminates the sample, the transmitted light is collected by a detection optical system and a camera. The sample is mounted on a translation and a rotation stage to be scanned and rotated around 360°. A stack of images is acquired in each angular position while scanning the sample (**a**). Scheme of the Optical Transfer Function of the microscope (**b**, upper panel). Acquiring multiple views is equivalent to rotating the OTF and sampling different spatial frequencies (**b**, lower panel). Reconstruction of the transverse section of a sample (*Arabidopsis thaliana*) using a single view (0°), 10 views (covering 60° in total) and 60 views (covering 360°), each with a spacing of 6°. The illumination and detection numerical aperture is NA = 0.13 (**c**). Scale bar: 50 µm.

The system required for BF multi-view reconstruction consists in a trans-illumination microscope in which the sample can be rotated over 360° (around the y axis in Fig. 1a) and translated along the optical axis (axis z in Fig. 1a). Like any wide-field microscope, the system presents a limited optical sectioning capability which is a consequence of the so called “missing cone” in the Optical Transfer Function (OTF)^33^ (Fig. 1b). For this reason, if we acquire a stack of images of the sample at different axial positions, the three-dimensional reconstruction that we obtain will have a limited axial resolution. We observe for example that the transverse section of a sample (an *Arabidopsis thaliana* root), presents such a poor axial resolution (Fig. 1c, left hand side) that different structures are indistinguishable, hindering any 3D analysis of the sample.

In order to obtain optical sectioning, we acquired multiple views of the specimen separated by an angle *Δα* and at each angle we collected a stack of images with sampling steps *Δz*. The different stacks were fused together to create a single reconstruction of the sample. We estimated the required number *N* of views by looking at the system OTF (Fig. 1b). Considering that, in paraxial approximation, the angle *θ* subtended by the OTF is approximately the system numerical aperture $NA = n sin\theta \approx n\theta$ (where *n* is the index of refraction), we chose to acquire the views at an angle step of $\Delta \alpha = \theta$ so that the maximum of each OTF overlapped with the minimum of the adjacent one. In this case, the number of acquired views resulted in $N = \frac{2\pi }{{\Delta \alpha }} = \frac{2\pi n}{{NA}}$. One could exploit the symmetry of the OTF and acquire only $\frac{N}{2}$ views around 180°, however in presence of scattering^34–36^ the acquisition of the sample around full 360° provided a more resolved reconstruction.

At each angle, in order to cover the specimen of thickness $L$*,* we acquired M images while translating the sample with a step *Δz*. Since the maximum axial cutoff frequency ^33^ of the microscope is $\Delta k_{z} = \frac{{NA^{2} }}{2\lambda n}$, following the Nyquist’s criterion we scanned the sample with an axial step $\Delta z = \frac{1}{{2 \Delta k_{z} }} = \frac{\lambda n}{{NA^{2} }}$. The number of acquisitions along *z* is given by $M = \frac{L}{\Delta z} = \frac{{L \cdot NA^{2} }}{\lambda n}$*.* We observe that the total number of acquired images $N_{TOT} = N \cdot M = \frac{2\pi \cdot L \cdot NA }{{ \lambda }}$ scales linearly with the numerical aperture, indicating that the approach is particularly suitable for numerical apertures and magnifications which are between those normally used in OPT and those used in high-resolution optical microscopy.

### Implementation in an optical tomography system and in a light sheet fluorescence microscope

The optical setup for BF reconstruction is similar to an OPT system^1,37,38^ except for two main adjustments: (1) a translation stage is required to scan the sample along the optical axis or to scan the detection optics along the sample; (2) the illumination numerical aperture should match that of the detection: $NA_{ILL} = NA_{DET}$. This second point is recommended to maximize the resolution of the microscope, which according to Abbe is $\frac{\lambda }{{NA_{ILL} + NA_{DET} }}$, and consequently to obtain reconstructions with higher-contrast.

**Figure 2:**
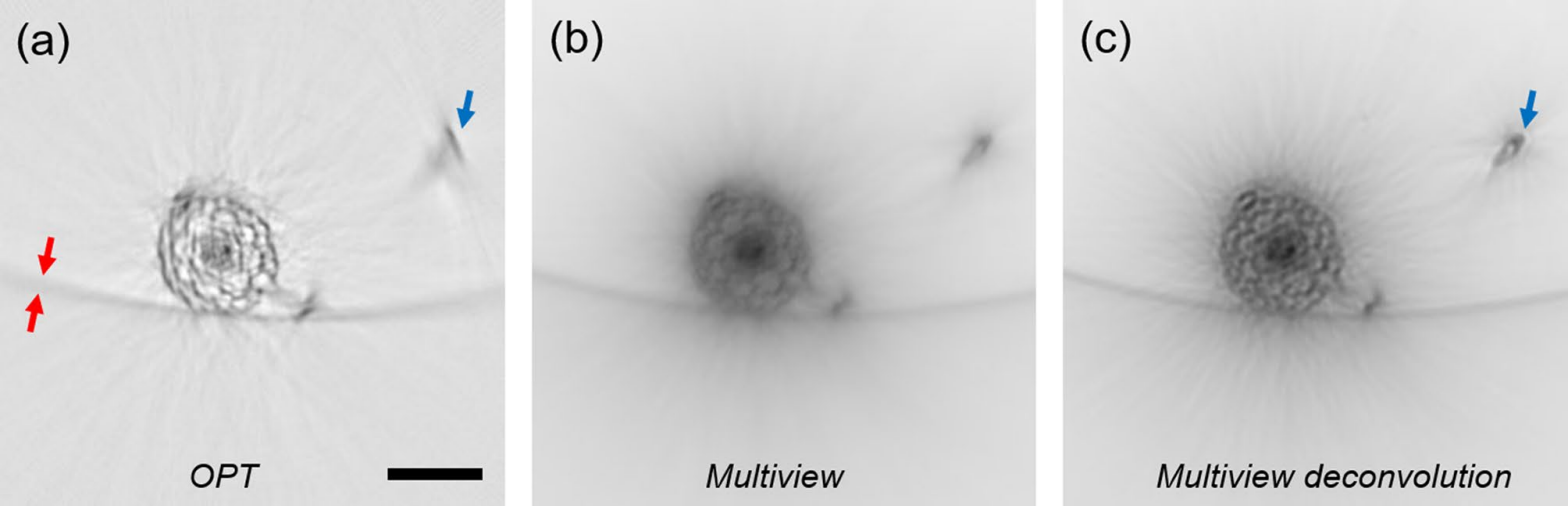
Reconstruction of a transverse section of an *Arabidopsis thaliana* root using Optical Projection Tomography (**a**), bright-field multi-view reconstruction (**b**) and bright-field multi-view deconvolution (**c**). For (**c**, **d**), 45 views around 360°, with 8° spacing were used. The presence of spatially variant artifacts in OPT is shown with the blue arrow. The red arrows indicate the thickness of the reconstructed plastic tube far from the rotation center of the OPT reconstruction. Scale bar: 100 µm.

On the other hand, the system required for BF reconstruction is also commonly present in multi-view LSFM microscopes, which allow translation and rotation of the specimen. Therefore, we performed our analysis on a LSFM microscope equipped with a Köhler illuminator for trans-illumination. In particular we used a 4× magnification (*NA* = 0.13) in the detection path: at this magnification and *NA*, the artifacts given by the diffraction are already present and significantly compromise the standard OPT results (Fig. 2a).

In this case, the theoretical number of acquired views is $N = \frac{2\pi n}{{NA}} \approx 48$ and the theoretical axial step is $\Delta z = \frac{\lambda n}{{NA^{2} }} \approx 30 \;\upmu {\text{m}}$ (here *λ* = 530 nm). For a sample of thickness *L* = 300 µm the number of steps results in *M ≈ *10. The results of the reconstruction performed for different values of *N* and *M* (Supplementary Fig. 1) confirm that the theoretical values are a good estimate: above *N* = 45 and *M* = 10 the increase in image contrast is negligible. The number of required views is therefore an order of magnitude smaller than the number of projections in standard OPT. Yet, the total amount of data remains practically constant because for each view a stack of images is required, but overall the acquisition time is comparable to that of standard OPT (1–5 min per sample).

The technique performs well also at higher *NA*. An example of reconstruction at *NA* = 0.3 with 10× magnification is shown in Supplementary Fig. 2. Here the diffraction artifacts in standard OPT are much stronger and a strategy for extending the depth of field^22,27,28^ would be in any case required. However, the focus of the present paper is to image an entire sample in a single measurement, which is demonstrated at lower magnification.

### Reduction of the diffraction artifacts

As described and characterized by Trull et al.^24^, the diffraction of light in OPT causes space-variant tangential blurring that increases with the distance from the rotation axis. We clearly observe these artifacts when imaging structures that are just hundreds of microns away from the rotation axis. This effect is shown in the completely distorted reconstruction (Fig. 2a, blue arrow) of a root hair of *Arabidopsis thaliana*, which is c.a 200 µm away from the rotation axis (located at the center of the OPT reconstructed section in Fig. 2a). In Fig. 2, the sample was embedded in a tube (see Material and Methods) that is visible in the OPT reconstruction due to the presence of a small amount of scattering which causes light attenuation. The size of the tube can be used as a benchmark to assess the presence of space-variant blurring. We observe that the higher the distance from the center of rotation, the more blurred the border of the tube is in the OPT reconstruction. When applying the multi-view approach, the sample is reconstructed correctly in the entire field of view, avoiding the diffraction artifacts (Fig. 2b). However, as expected, by simply overlapping (sum) the views acquired at different angles, the image is overall blurred. In order to increase the quality of the reconstruction we used a deconvolution approach based on the Wiener or Lucy Richardson methods (see Material and Methods). In this way, the contrast and resolution are significantly improved leading to the observation of the Arabidopsis section at single cell detail (Fig. 2c). This improvement comes at the expense of higher noise, and in order to fully exploit the capabilities of the method a systematic study on deconvolution and regularization should still be carried out. It is worth noting that in case of highly diffusive samples the technique would be able to reconstruct only the outermost part of the specimen with artifacts given by the diffusion of light. Therefore, the method should be applied to translucent samples (as it is the case for zebrafish embryos and *Arabidopsis* roots), chemically cleared samples, or it should be combined with more advanced methods to reduce the effect of diffusion^34,35,39^.

### Search for the center of rotation

To accurately reconstruct the data, the position of the rotation axis must be determined at pixel resolution. We propose a straightforward method based on two processing steps to locate the rotation axis, both in *x* and *z*, assuming that the latter is parallel to the *y* axis (as in Fig. 1a).

**Figure 3:**
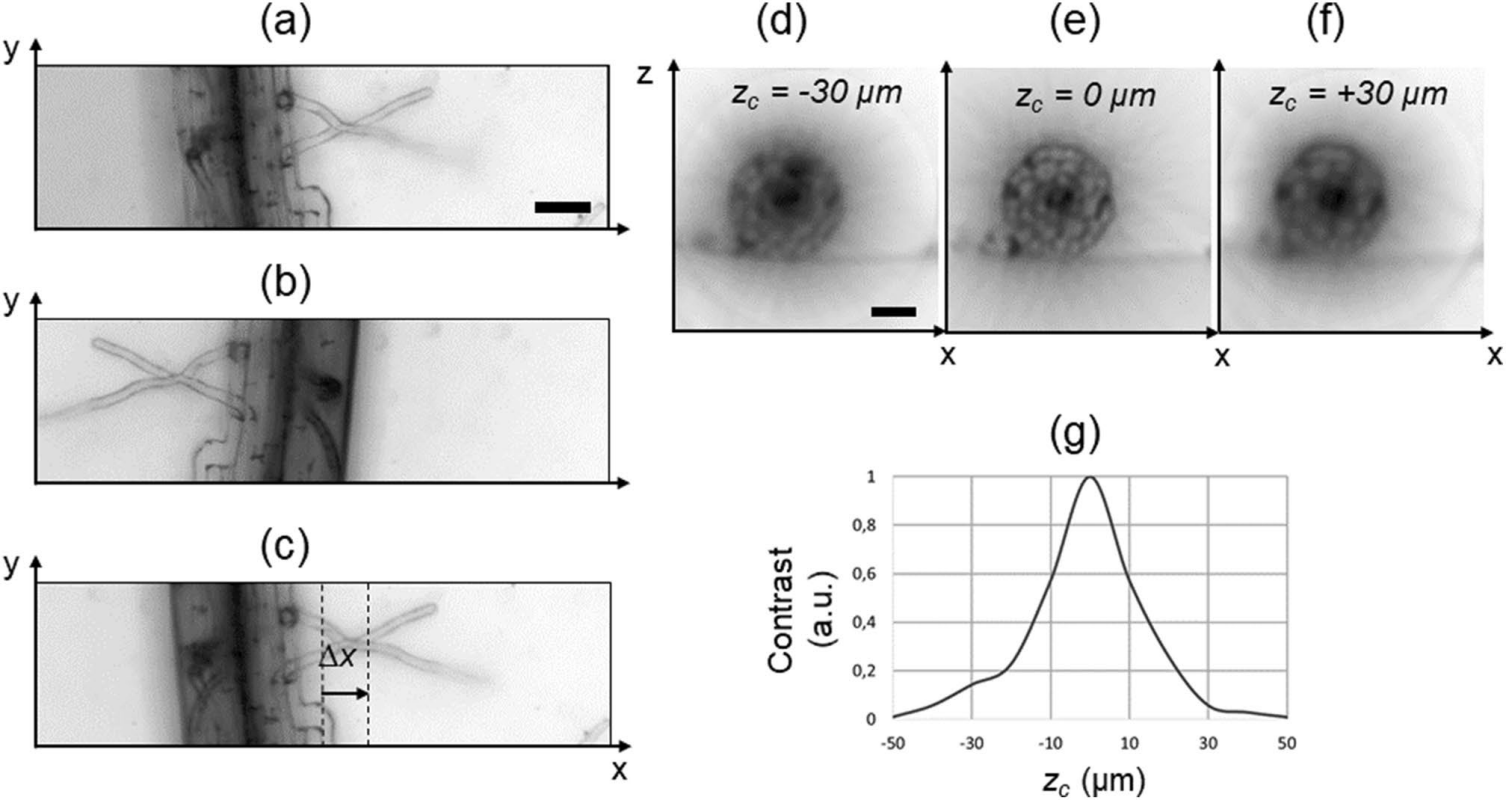
Search of the rotation axis. Minimum intensity projection (each pixel shows the minimum value of the stack calculated along the z direction) of the stack acquired at the angle 0° (**a**). Minimum intensity projection of the stack acquired at the angle 180° (**b**). The image shown in b, is flipped horizontally and translated to overlap the image in a. The distance Δx/2 indicates the position of the rotation axis from the image center, along the horizontal direction (**c**). Blurring artifacts arising from incorrectly identified axial position of the rotational axis z_C_ (**d**–**f**). The contrast of a series of test reconstructions (**g**) has a maximum at the position corresponding to the correct rotational axis (z_C_ = 0). Scale bars: 50 µm.

First, two stacks of images are acquired at opposite angles (e.g. 0° and 180°) and the two corresponding projections are created (mean intensity projection or minimum intensity projection, as shown in Fig. 3a, b). One of the two projections is flipped along the *x* axis and translated to overlap to the other using an image registration algorithm: in order to overlap the two images, the second projection is translated by a distance *Δx* (Fig. 3c). The location *x*_*C*_ of the rotation axis is then calculated as the sum of semi-width of the image and the distance *Δx/*2*.*

Secondly, to determine the location *z*_*C*_ of the rotational axis along the *z* axis, we adopted an approach typically used in OPT^38^. We select a single transverse section of the sample (in a certain *y* location), reconstruct the section assuming different *z*_*C*_ values and calculate the contrast of each reconstruction (Fig. 3d–g), as described in Material and Methods. The reconstructed image that has the highest contrast is the closest to the ideal reconstruction, as it is the least blurred. The corresponding *z*_*C*_ position is considered to be that of the rotation axis.

### *In-vivo* imaging

**Figure 4:**
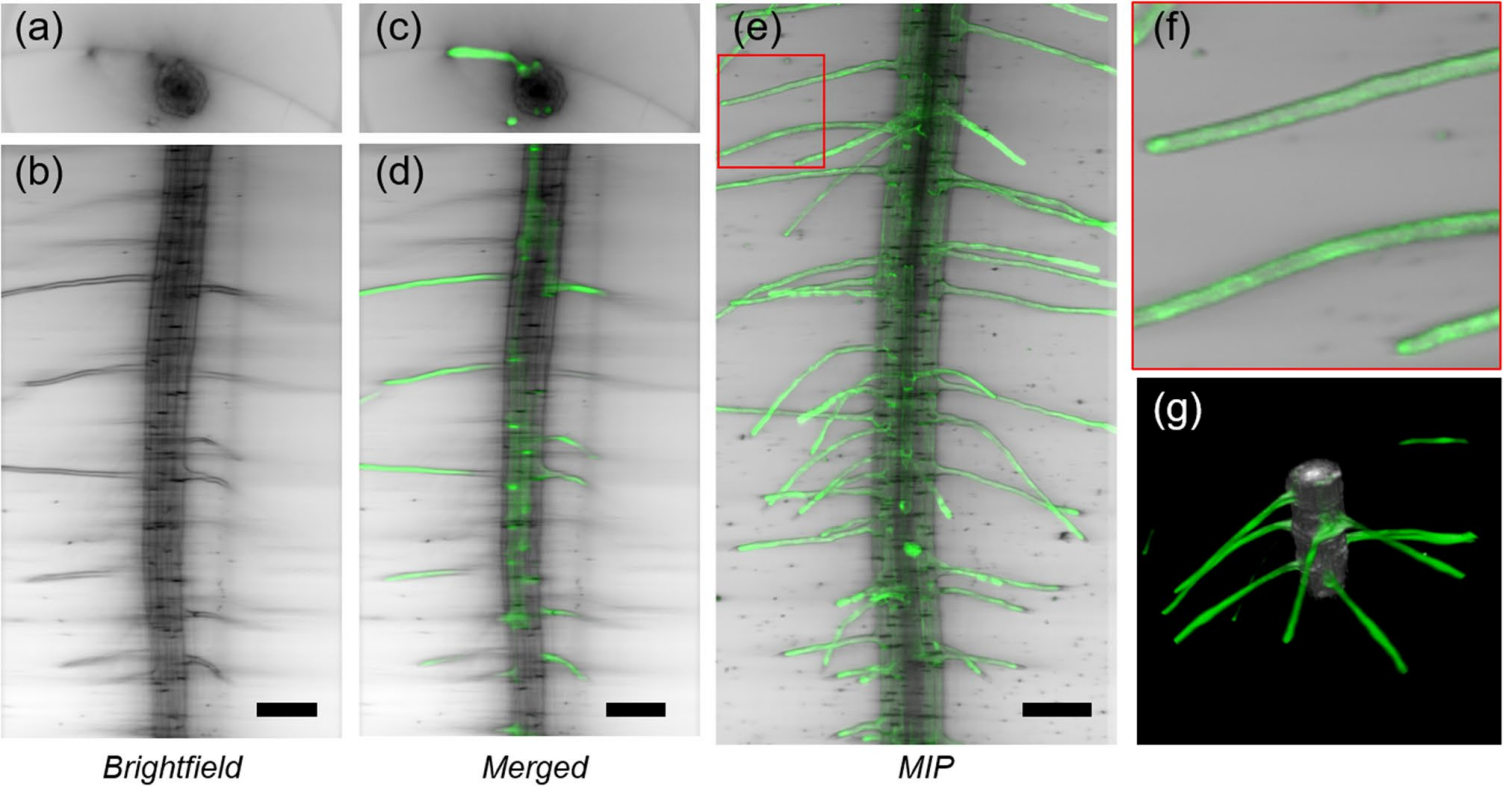
Three-dimensional imaging of a transgenic pEXP7:YC3.6 *Arabidopsis thaliana* seedling. Transverse (**a**) and lateral (**b**) sections of root tip (mature zone) acquired with bright-field multi-view reconstruction. Transverse (**c**) and lateral (**d**) sections acquired with bright-field reconstruction (grey) and LSFM (green). Maximum intensity projection of the LSFM stack, combined with the minimum intensity projection of the bright-field reconstruction (**e**). Detail of panel e, showing the outgrowing part of the root-hair epidermal cells (**f**). Three-dimensional rendering of the reconstructed bright-field volume, combined with LSFM. Scale bar: 100 µm.

Using BF reconstruction, we managed to acquire a large volume of the biological sample in a single measurement. We tested the method in vivo with *Arabidopsis thaliana* and zebrafish (*Danio rerio*) samples. In the root of *Arabidopsis* seedlings, the reconstruction can be extended to a large portion of the root including the root hairs, which are long tubular-shaped outgrowths from root epidermal cells (Fig. 4 and Supplementary Fig. 3). One key feature of the technique is to provide isotropic resolution over the entire reconstructed volume. In addition, the method is label-free, since the BF reconstruction is based on trans-illumination. In living samples, the contrast is given by the attenuation of light, primarily due to scattering and absorption. Therefore, while on the one hand the staining is not required for imaging, on the other hand, since low-power light sources are used for trans-illumination, the measurements induce minimum photo-toxicity to the biological sample. These features make the technique an ideal tool for assessing the anatomy of the sample *in-vivo*.

BF reconstruction can be easily implemented in a multi-view light sheet microscope, complementing the high-resolution fluorescence reconstruction obtained with LSFM. In particular, we acquired images from transgenic *Arabidopsis* seedlings expressing the Cameleon YC3.6^40^ under the control of *At*EXP7 promoter (pEXP7:YC3.6) which directs root hair-specific expression (trichoblast cells) of the fluorescent sensor^41^. This acquisition shows that by combining LSFM with BF reconstruction the fluorescence can be precisely localized in the context of a single tissue sections (Fig. 4c, d) or in the entire volume (Fig. 4e). It is worth noting that the BF reconstruction is performed with a LED illumination at *λ* = 530 nm, close to the emission wavelength of GFP, avoiding any chromatic aberration (Fig. 4f).

**Figure 5:**
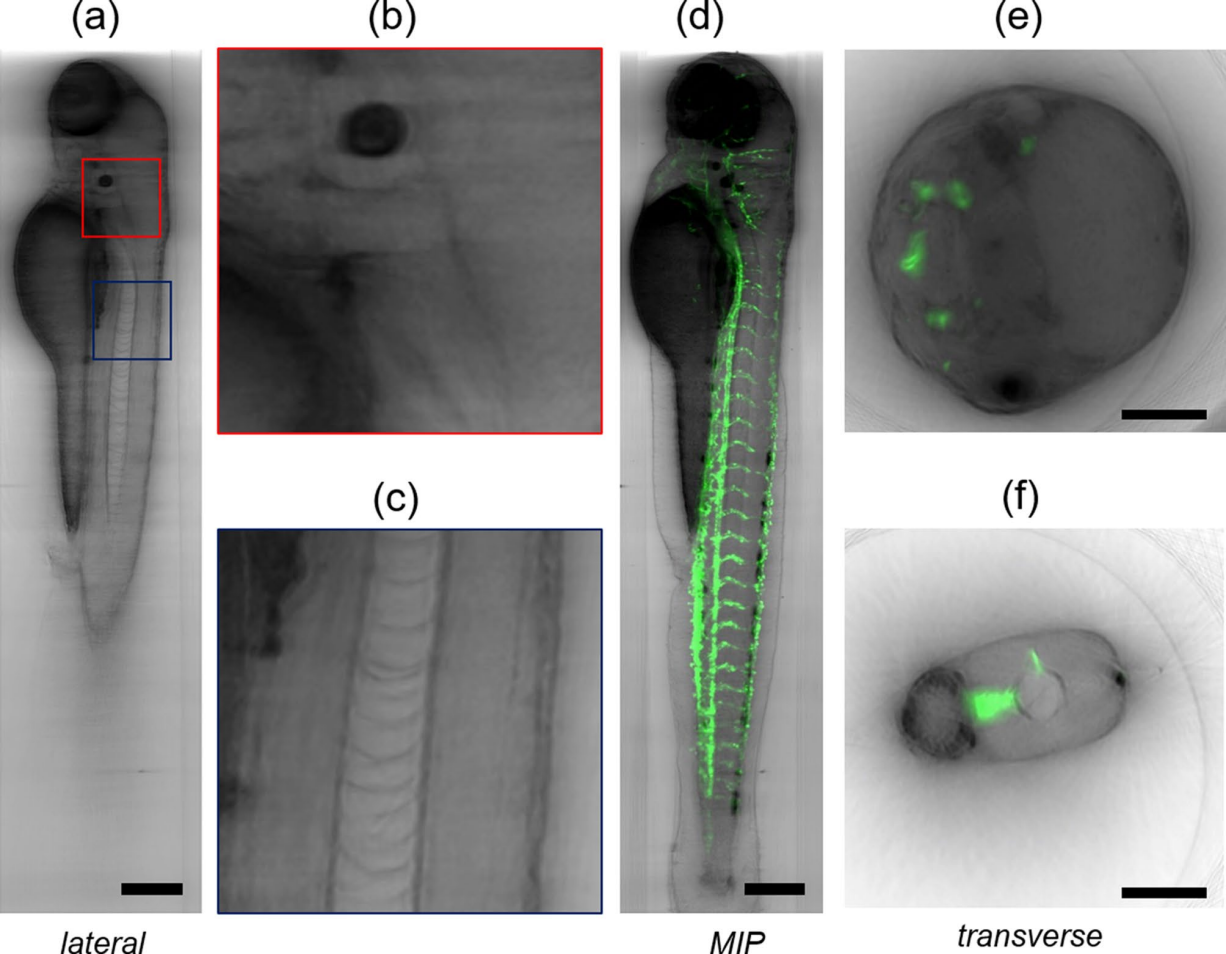
Sagittal slice of a transgenic Tg(kdrl:GFP) zebrafish (4 dpf) visualized with bright-field multi-view reconstruction (**a**). Details of the sample showing a portion of the head (**b**) and of the zebrafish notochord (**c**). Minimum intensity projection of the bright-field reconstruction (grey) overlapped with the maximum intensity projection of the LSFM data (green). The bright-field reconstruction shows the whole zebrafish anatomy while LSFM shows the labelled vasculature (**d**). Transverse section of the sample in the head (**e**) and tail (**f**), showing the bright-field reconstruction (grey) and LSFM (green). Scale bars: 100 µm.

Measurements of zebrafish embryos demonstrate that the whole organism can be acquired in a single experiment. Again, the technique offers the possibility to observe several organs (e.g. notochord, yolk, eye, brain) in single sections (Fig. 5a–c and Supplementary Fig. 4) and within the entire sample anatomy. The use of 4 days post fertilization (dpf) transgenic *VEGFR2:GFP* zebrafish embryos that express green fluorescent protein under the control of the vascular endothelial promoter VEGFR2/KDR [*Tg(kdrl:GFP)*]^42^, confirms that the combination of LSFM and BF reconstruction is a suitable tool to localize a sparse fluorescence signal in the tissue (Fig. 5d–f).

In summary we have shown that three-dimensional reconstructions of unstained samples can be achieved in multi-view microscopes, with isotropic resolution, typical of OPT, but without the diffraction artifacts that affect OPT reconstruction. The bright-field multi-view reconstruction provides a comprehensive picture of zebrafish and *Arabidopsis thaliana* anatomy, including organs that are usually not labelled and therefore not observable. The bright-field contrast nicely complements the fluorescence contrast observable with LSFM, allowing correlative fluorescent protein expression and anatomical visualization.

## Material and methods

### Fish lines and sample preparation

Zebrafish AB strains obtained from the Wilson lab (University College London, London, UK) were maintained at 28 °C on a 14 h light/10 h dark cycle. The zebrafish transgenic *Tg(kdrl:GFP)* was used for fluorescence imaging. Embryos were collected by natural spawning, staged according to Kimmel and colleagues, and raised at 28 °C in fish water (Instant Ocean, 0.1% Methylene Blue) in Petri dishes, according to established techniques. After 24 hpf, to prevent pigmentation 0.003% 1-phenyl-2-thiourea (PTU, Sigma-Aldrich, Saint Louis, MO, USA) was added to the fish water. Embryos were washed, dechorionated and anaesthetized, with 0.016% tricaine (Ethyl 3-aminobenzoate methanesulfonate salt; Sigma-Aldrich, before acquisitions. During imaging, the fish were restrained in FEP (Fluorinated ethylene propylene) tubes^37^.

#### Plants

*Arabidopsis thaliana* seedling preparation was carried out accordingly to Candeo et al.^43,44^. and Romano Armada et al.^45^. Briefly, seeds of *Arabidopsis thaliana* Col-0 transformed with pEXP7:YC3.6 were surface sterilized by vapour-phase sterilization and plated on MS/2 medium supplemented with 0.1% (w/v) sucrose, 0.05% (w/v) MES, pH 5.8 adjusted with KOH and solidified with 0.8% (w/v) plant agar (Duchefa, The Netherlands). After stratification at 4 °C in the dark for 2–3 days, seeds were transferred to the growth chamber with 16/8 h cycles of light (70 µmol m^−2^ s^−1^) at 24 °C.

After seedling germination and fluorescence inspection, the germinated fluorescent seeds were moved from the plate to the top of FEP tubes filled with gel (prepared accordingly to Candeo et al.,^43^ and Romano Armada et al.^45^, using sterilized pliers and without clamping them, so the plantlets could grow inside the filled tubes. The tubes were transferred to a tip box that was finally filled with MS/2 liquid medium without sucrose and sealed to avoid contamination. The mounting procedure and the special illumination and detection configuration of OPT-LSFM allowed the seedlings to be held from the top of the chamber in a vertical position, with the roots growing directly in the jellified medium inside the transparent tubes. To mount the tubes with the plant in the imaging chamber, we used the custom holder reported in Candeo et al.^43^. When plants were ready to be imaged, we plugged the pipette tip with the tube into the holder, and quickly moved it to the imaging chamber, fixing it on a rotation and translation stage for the sample positioning.

#### Setup and acquisition protocol

A Köhler illuminator is used for trans-illumination of the sample. The light source is a LED (Thorlabs M530L2) emitting at 530 nm. The transmitted light is collected by a microscope objective lens (Nikon *M* = 4×*, NA*_*DET*_ = 0.13 or Olympus *M* = 10×,* NA*_*DET*_ = 0.3) and tube lens to form the image of the sample on a sCMOS camera (Hamamatsu, Flash 4.0). A long pass filter at 500 nm (Thorlabs FEL0500) is placed between the objective lens and the tube lens, suitable for the collection of 530 nm illumination and also for GFP fluorescence detection. The numerical aperture of the Köhler illuminator is matched to that of the detection (*NA*_*ILL*_ = *NA*_*DET*_). The sample is immersed in a cuvette filled with medium from the top, and is translated along the z-axis with a linear stage (Physik Instrument, M-404.1PD) and rotated with a rotation stage (Physik Instrument M-660.55). A manual translator (Thorlabs, ST1XY-S/M) is mounted on the rotation stage to move the sample on the *xz* plane and position it in the proximity of the rotation axis. This ensures that the specimen is within the field of view at all the acquisition angles. The sample holder and the pre-alignment protocol are described in Bassi et al.^22^. The experiments are performed with a custom-made light sheet microscope^46^. For LSFM illumination, a solid-state laser emitting at 473 nm (CNI, MBL-FN-473) is used. The laser beam is expanded to a diameter of 7 mm and split into two portions in order to illuminate the sample from opposite sides: two cylindrical lenses (Thorlabs, LJ1703RM-A, f = 75 mm) are used to create a light sheet made by two counter-propagating beams across the sample. The illumination is perpendicular to the detection path, with the light-sheet formed in the focal plane of the detection objective lens. LSFM and bright-field reconstruction share the same detection system and the acquisition protocol is the same for the two modalities.

1. The sample is translated along the optical axis and images are continuously acquired by the camera. The velocity of the linear stage is synchronized with the acquisition so that every captured image corresponds to a linear step of *Δz.*
2. Multimodal acquisition (bright-field/fluorescence) is performed alternating the LED (for trans-illumination) and the laser (for LSFM). After each axial scan required for bright-field acquisition, a second axial scan is repeated for LSFM acquisition. To this end two custom-made mechanical shutters are used to alternate the illuminations.
3. The sample is rotated by $\Delta \alpha$ and the previous points are repeated for multiview acquisition. Typically the angle $\Delta \alpha$ is chosen so to have a full 360° rotation after *N* + 1 rotation steps.
4. For time lapse acquisitions, the previous points are repeated, typically every 10 min.

#### Data processing

The reconstruction consists in a multi-view fusion of the data acquired at different angles. Data processing was performed in Python; a sample code is available on GitHub (https://github.com/andreabassi78/BrightfieldMultiviewReconstruction).

The x location of the center of the rotation axis is found by registering two opposite views projections. The registration is performed using the Python module scikit-image (register_translation) to determine the distance *Δx* (Fig. 3c).

Then, stacks acquired at the different angles are resliced into planes (*xz*) which are orthogonal to the rotation axis. The z location of the center of rotation is found by maximizing the contrast of the reconstruction at different possible z_C_. The contrast is calculated as the energy (sum) of all the frequency components obtained by numerically Fourier transforming (fft2 function in the Python module numpy) the reconstruction, excluding the DC component.

This approach assumes that the rotation axis is perfectly perpendicular to the optical axis of the detection objective. If this is not the case, we suggest to repeat the procedure at two or more y locations and then linearly interpolate the values of x_C_ and z_C_ for all the considered y values.

Once the rotation axis is defined we process each plane independently, following an approach that has been proven to work properly in multi-view reconstruction of LSFM data in zebrafish^47^ and offers the possibility to be accelerated using graphics processing units (GPU), potentially providing the results in real time. Each xz plane is rotated around (x_C_, z_C_), by its angle view, and summed to the other views. In total, N different views are summed (N is the number of acquired image stacks around 360°) and the resulting image is divided by N. This mean image is the reconstructed plane. If deconvolution is applied, each xz plane is de-convolved before the sum. For deconvolution we used the theoretical point spread function (PSF) of the microscope. This is generated considering the numerical aperture of the lens, creating the corresponding Ewald sphere^33^, and calculating the absolute value squared of its 3D Fourier transform. For plane by plane processing, a two dimensional PSF is extracted from its three dimensional counterpart by slicing it in its central section. Deconvolution is then performed using the Wiener or Lucy Richardson filtering in the Python package scikit-image.

The results are visualized using Fiji.

## Methods guidelines

All methods were carried out in accordance with relevant guidelines and regulations. Zebrafish (*Danio rerio*) were maintained at the zebrafish facility, University of Milan, Via Celoria 26—20133 Milan, Italy (Aut. Prot, n. 295/2012-A—20/12/2012 for the breeding, growth and use of zebrafish, released by the Azienda di Tutela della Salute, ATS Città metropolitana di Milano). All experimental procedures were performed according to the international (EU Directive 2010/63/EU) and national guidelines (Italian decree No 26 of the 4th of March 2014). Embryos were staged and used until 5 days post fertilization, a time windows in which zebrafish is not considered an animal model according to national guidelines (Italian decree No 26 of the 4th of March 2014). All procedures to minimize stress and pain of the embryos were applied. Embryos were anaesthetized with 0.016% tricaine (Ethyl 3-aminobenzoate methanesulfonate salt, Sigma-Aldrich) before proceeding with experimental protocols.

## Supplementary information

Supplementary Information.
