# Supplementary material for: Three-dimensional bright-field microscopy with isotropic resolution based on multi-view acquisition and image fusion reconstruction
Source: Sci Rep. 2020 Jul 29;10:12771. doi: 10.1038/s41598-020-69730-4 (PMC7392767; doi:10.1038/s41598-020-69730-4)
Supplement: Supplementary file 1 — Supplementary Information. [file 41598_2020_69730_MOESM1_ESM.docx]

**Three-dimensional bright field microscopy with isotropic resolution based on multi-view acquisition and image fusion reconstruction**

Supplementary Figures

Gianmaria Calisesi^1^, Alessia Candeo^1^, Andrea Farina^2^, Cosimo D’Andrea^1^, Vittorio Magni^1^, Gianluca Valentini^1,2^, Anna Pistocchi^3^, Alex Costa^4^, Andrea Bassi^1,2*^

1. Dipartimento di Fisica, Politecnico di Milano, piazza Leonardo da Vinci 32, 20133 Milano, Italy

2. Istituto di Fotonica e Nanotecnologie, Consiglio Nazionale delle ricerche, piazza Leonardo da Vinci 32, 20133 Milano, Italy

3. Dipartimento di Biotecnologie Mediche e Medicina Traslazionale, Università degli Studi di Milano, via Fratelli Cervi 93, 20090 Segrate, MI, Italy

4. Dipartimento di Bioscienze, Università degli Studi di Milano, via Celoria 26, 20133 Milano, Italy


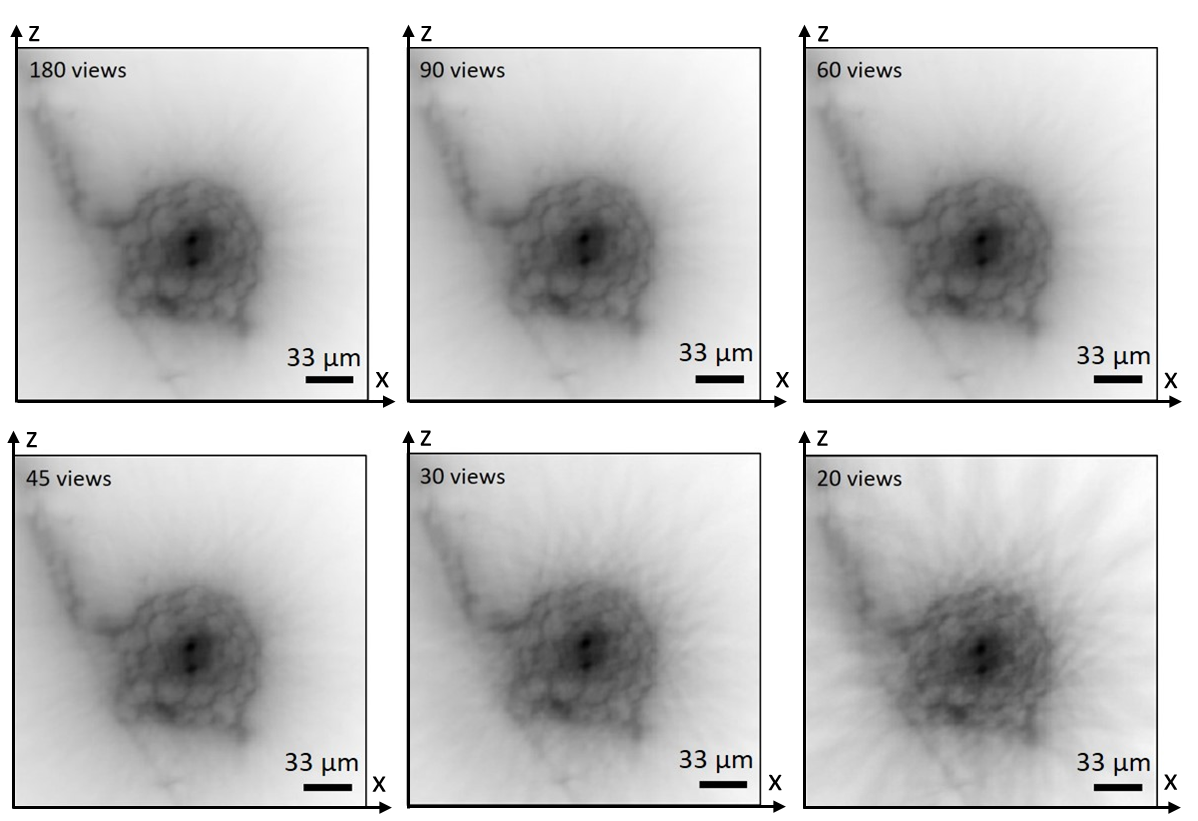


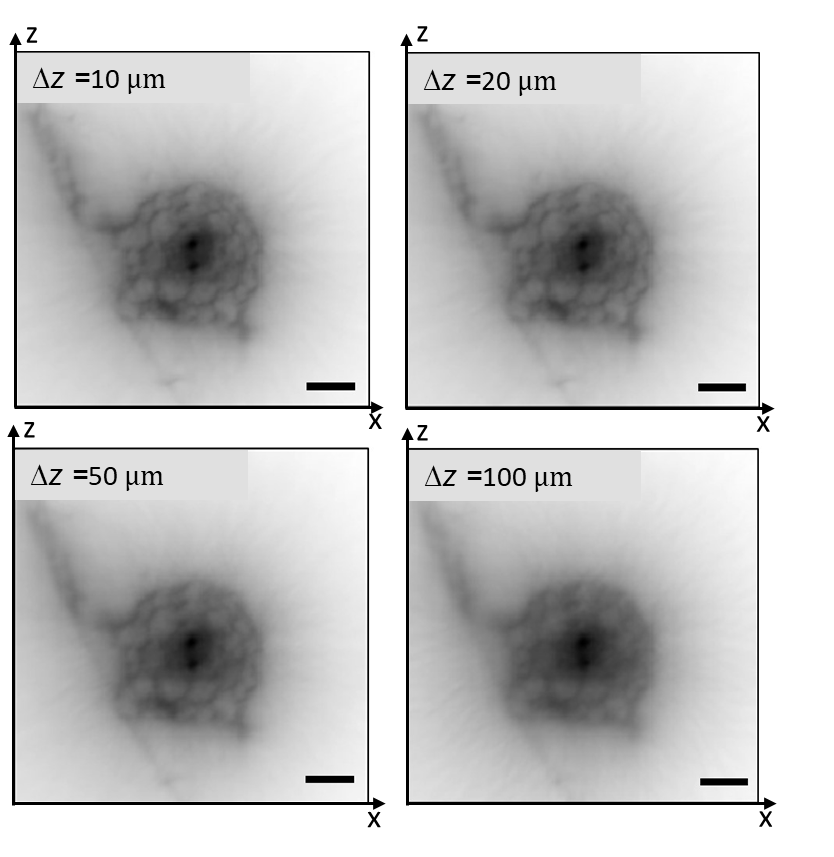


**Supplementary Figure 1**. Reconstruction of a transverse section of an Arabidopsis thaliana root (mature zone) with different acquisition parameters. Stacks acquired with a 4X, NA = 0.13 objective lens. Scale bars: 33 µm in all panels

Upper panel: Reconstruction obtained using the same axial step Δz = 20µm and different number of views. The angle step Δα is 360° divided by the number of acquired views N, indicated in the figure panels.

Lower panel: Reconstruction obtained with the same angular step Δα = 6° and different axial steps. The number of acquired images M for each axial scan is given by the scanned thickness (c.a. 300 µm) divided by the axial step Δz, indicated in the figure panels.


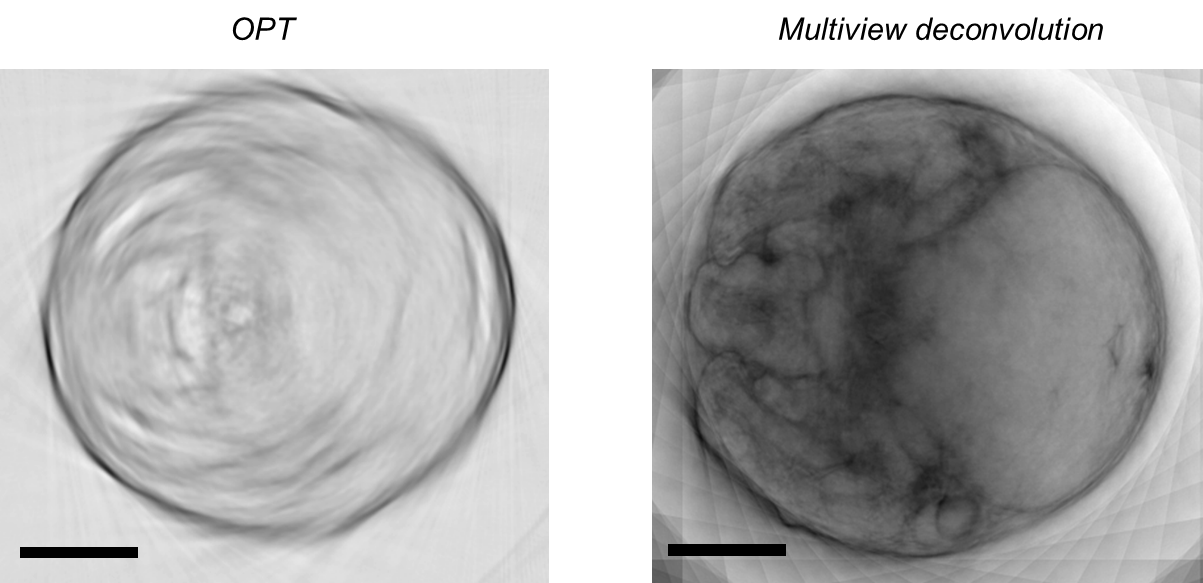


**Supplementary Figure 2**. Comparison of the reconstruction of a zebrafish (3.5dpf) transverse section with OPT (left hand side) and multiview reconstruction (right hand side). Illumination and detection are performed with a 10X objective, NA=0.3. The number of acquired angles was N=20 with an angle step of Δα=18°. The axial step was Δz=8 µm. Scale bar: 100 µm


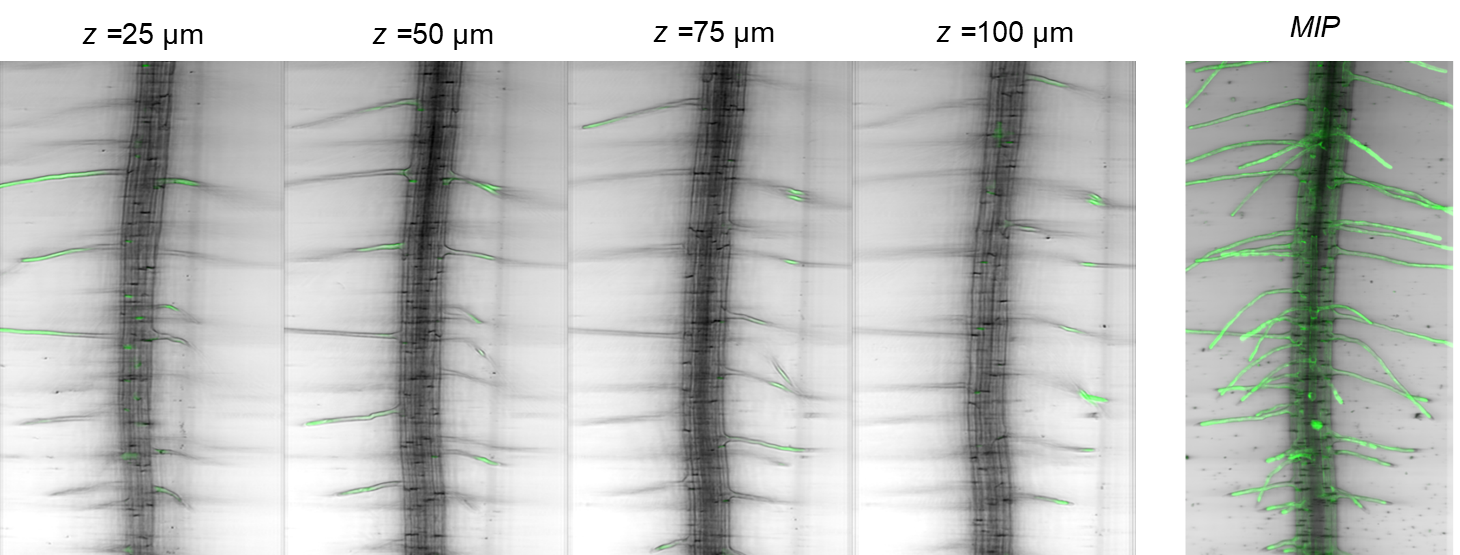


**Supplementary Figure 3**. Lateral slices of *Arabidopsis thaliana* root reconstructed at 25 µm steps one from the other. Brightfield reconstruction (grey) is overlapped with LSFM reconstruction of the labelled cells. The position z=0 µm corresponds to the edge of the root. The panel on the right hand side is a maximum intensity projection of the LSFM stack, combined with the minimum intensity projection of the bright-field reconstruction.


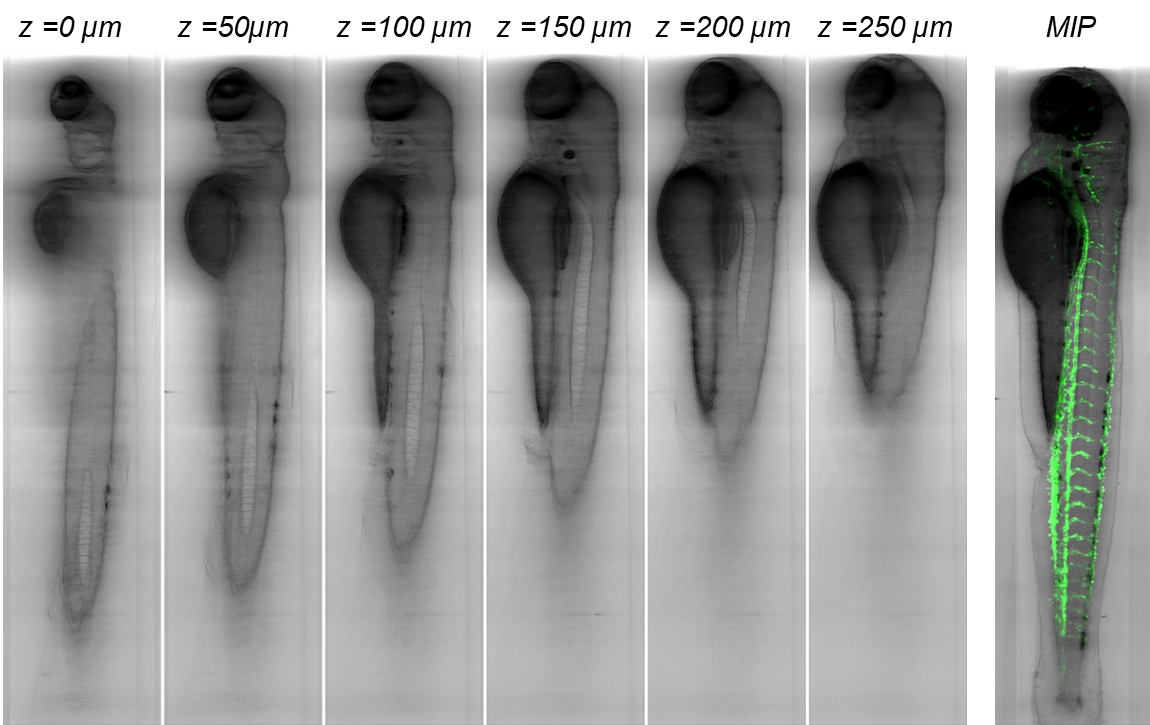


**Supplementary Figure 4**.

Sagittal slices of a transgenic Tg(kdrl:GFP) zebrafish (4 dpf) visualized with bright-field multi-view reconstruction. The slices are shown at 50 µm steps.

The panel on the right hand side is a minimum intensity projection of the bright-field reconstruction (grey) overlapped with the maximum intensity projection of the LSFM data (green). The bright-field reconstruction shows the whole zebrafish anatomy while LSFM shows the labeled vasculature.
